# Policaptil Gel Retard® significantly reduces body mass index and hyperinsulinism and may decrease the risk of type 2 diabetes mellitus (T2DM) in obese children and adolescents with family history of obesity and T2DM

**DOI:** 10.1186/s13052-015-0109-7

**Published:** 2015-02-15

**Authors:** Stefano Stagi, Elisabetta Lapi, Salvatore Seminara, Paola Pelosi, Paolo Del Greco, Laura Capirchio, Massimo Strano, Sabrina Giglio, Francesco Chiarelli, Maurizio de Martino

**Affiliations:** Department of Health Sciences, University of Florence, Anna Meyer Children’s University Hospital, Florence, Italy; Human Genetic Unit, Anna Meyer Children’s University Hospital, Florence, Italy; Paediatric Unit, Mugello’s Hospital, Borgo San Lorenzo, Florence, Italy; Department of Paediatrics, University of Chieti, Chieti, Italy

**Keywords:** Obesity, Children, Type 2 diabetes, Metabolic syndrome, Policaptil Gel Retard®

## Abstract

**Background:**

Treatments for childhood obesity are critically needed because of the risk of developing co-morbidities, although the interventions are frequently time-consuming, frustrating, difficult, and expensive.

**Patients and methods:**

We conducted a longitudinal, randomised, clinical study, based on a per protocol analysis, on 133 obese children and adolescents (n = 69 males and 64 females; median age, 11.3 years) with family history of obesity and type 2 diabetes mellitus (T2DM). The patients were divided into three arms: Arm A (n = 53 patients), Arm B (n = 45 patients), and Arm C (n = 35 patients) patients were treated with a low-glycaemic-index (LGI) diet and Policaptil Gel Retard®, only a LGI diet, or only an energy-restricted diet (ERD), respectively. The homeostasis model assessment of insulin resistance (HOMA-IR) and the Matsuda, insulinogenic and disposition indexes were calculated at T_0_ and after 1 year (T_1_).

**Results:**

At T_1_, the BMI-SD scores were significantly reduced from 2.32 to 1.80 (p < 0.0001) in Arm A and from 2.23 to 1.99 (p < 0.05) in Arm B. *Acanthosis nigricans* was significantly reduced in Arm A (13.2% to 5.6%; p < 0.05), and glycosylated-haemoglobin levels were significantly reduced in Arms A (p < 0.005). The percentage of glucose-metabolism abnormalities was reduced, although not significantly. However, the HOMA-IR index was significantly reduced in Arms A (p < 0.0001) and B (p < 0.05), with Arm A showing a significant reduction in the insulinogenic index (p < 0.05). Finally, the disposition index was significantly improved in Arms A (p < 0.0001) and B (p < 0.05).

**Conclusions:**

A LGI diet, particularly associated with the use of Policaptil Gel Retard®, may reduce weight gain and ameliorate the metabolic syndrome and insulin-resistance parameters in obese children and adolescents with family history of obesity and T2DM.

## Introduction

Childhood obesity, which is recognised as a major public health issue, has been increasing worldwide during the last several decades [1]. More than one-third of children and adolescents are reportedly at risk of being overweight or obese in Italy [2] and in many countries of Europe [3-5]. The need for evidence-based treatment recommendations is a critical healthcare issue because obese children and adolescents are at risk for developing many co-morbidities observed in obese adults. This increase in weight, as well as the decrease in physical activity and cardiorespiratory fitness levels [6], are in direct proportion to an increasing prevalence of impaired glucose tolerance (IGT) [7], metabolic syndrome (MetS) [8,9], and type 2 diabetes (T2DM) [10]. Nevertheless, many data show lower rates in Europe compared to North American data [11].

The treatment of childhood obesity is frequently time-consuming, frustrating, difficult, and expensive [12]. Many interventions have focused on changing individual behaviour to prevent excessive childhood weight gain; however, this strategy has generally led only to short-term improvements, if any, in obesity and its related risk factors [13]. Obesity is challenging to treat because of multiple physiological, behavioural, and cultural feedback loops [14].

According to the energy balance model, a change in body weight is equal to the energy intake minus the energy expenditure. However, many studies have shown that energy input and expenditure are interdependent and are regulated at several levels, which suggests that a more intricate model operates to regulate energy balance and to maintain body weight [15].

Dietary recommendations are a central component of any comprehensive weight-loss programme and various alternative dietary interventions, such as energy restricted diets (ERD), reduced carbohydrate diets, and low-glycaemic-index (LGI) diets, have been proposed [16]. An ERD is the conventional treatment for obesity; however, the results indicate that it is not easy to follow and that it is difficult to achieve and maintain weight loss. Satiety is inversely related to the glycaemic and insulinaemic responses, and diets that reduce the insulin response may better promote long-term weight loss by decreasing hunger [15].

The purpose of this study was to compare the effects of a LGI diet in conjunction with Policaptil Gel Retard®, a new complex of polysaccharide macromolecules slowing the rate of carbohydrate and fat absorption, and LGI and ERD diets alone on the glucose metabolism parameters in three cohorts of obese children and adolescents.

## Subjects and methods

This longitudinal, randomised, clinical study, based on a per protocol analysis, enrolled 150 obese patients (n = 75 males, 75 females; median age at the onset of the study, 11.4 years; age range, 8.0–13.8 years) with familial forms of obesity and T2DM, consecutively evaluated between February 2012 and February 2014 at Anna Meyer Children’s University Hospital in Florence, and at Mugello’s Hospital in Borgo San Lorenzo, Florence, Italy.

The study was approved by the competent Ethics Committees and was conducted according to the Declaration of Helsinki. Informed written consent was obtained from the subjects’ parents before their participation in the study.

### Study design

**Figure 1:**
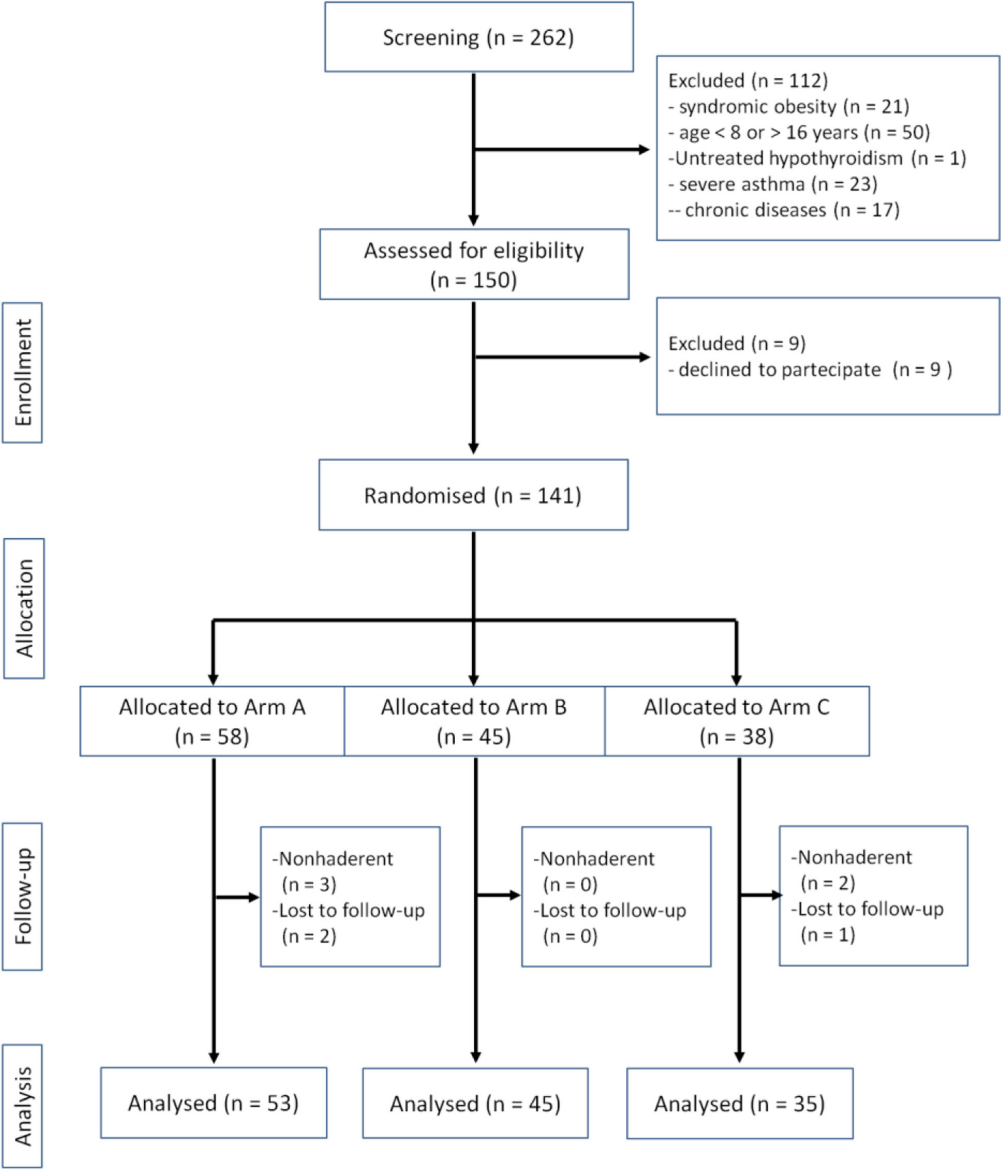
**Summary of patient flow diagram.** Individuals with obesity and family history for obesity and T2DM. The patients in Arm A were treated with a LGI diet and Policaptil Gel Retard® during the study; the patients in Arm B were treated with a LGI diet alone, whereas the patients in Arm C were treated only with an ERD of reduced carbohydrates and fats.

The children and adolescents were recruited before therapeutic intervention began (T_0_) and were monitored for 1 year (T_1_) (median, 11.9 months; range, 8.1–13.3 months). These subjects were randomly divided into three groups that were matched according to age, sex, and body mass index (BMI) (Figure 1).

The exclusion criteria were as follows: patients younger than 8 years and older than 16 years, a pre-existing T2DM, Cushing syndrome, untreated hypothyroidism, severe asthma, use of medications known to promote weight gain or loss, obesity-associated genetic syndromes, chronic diseases, or systemic inflammation [17].

Randomisation was performed using a computer-generated random number table with 1.3:1:1 randomisation (Arm A, 60 subjects; Arm B, 45 subjects; Arm C, 45 subjects). Auxological data and laboratory measurements were obtained at baseline and at 3–6 months and 12 months.

Of the 150 patients initially recruited to participate in this study, 9 (6.0%; n = 2 males, 7 females) declined to participate (Figure 1). However, 8 (n = 4 males, 4 females) dropped out for various reasons (non-compliance, lost to follow up, etc.), and were excluded from the study: 5 subjects belonged to Arm A (3 dropped out for non-compliance, 2 for loss to follow up) and 3 subjects belonged to Arm C (2 dropped out for non-compliance, 1 for loss to follow up); all of the subjects in Arm B remained in the study. The final count, 133 subjects (87.2%; 69 males, 64 females; median age at the onset of the study, 11.3 years; age range, 8.0–13.5 years) completed the study (Figure 1). However, to avoid the possibility of misleading artifacts that can arise in a per protocol analysis, such as non-compliance or drop-outs, we evaluated the results at T_0_ and T_1_ also by an intention-to-treat (ITT) analysis (141 subjects 73 males, 68 females; median age at the onset of the study, 11.4 years; age range, 8.0–13.6 years) (Table 1).

**Table 1:** **Primary baseline anthropometric, clinical, and biochemical features of the study groups**

	Arm A*	**Arm A°**	Arm B*°	Arm C*	**Arm C°**
Age, years (range)	11.7 (8.0–13.5)	11.7 (8.0–13.6)	12.0 (8.2–13.5)	11.8 (8.1–13.1)	11.8 (8.0–13.2)
N° and sex (male/female)	53 (27/26)	58 (29/29)	45 (23/22)	35 (19/16)	38 (21/17)
Tanner stage					
Prepubertal, n (%)	30 (56.6)	33 (56.9)	24 (53.3)	19 (54.3)	21 (55.3)
Pubertal, n (%)	23 (43.4)	25 (43.1)	21 (46.6)	16 (45.7)	17 (44.7)
Prepubertal/pubertal ratio, n (%)	30/23 (56.6/43.4)	33/25 (56.9/43.1)	24/21 (53.3/46.6)	19/16 (54.3/45.7)	21/17 (55.3/44.7)
BMI, SDS	2.32 ± 0.53	2.33 ± 0.57	2.23 ± 0.57	2.27 ± 0.74	2.29 ± 0.77
*Acanthosis nigricans*, n (%)	7 (13.2)	8 (13.8)^1^	6 (13.3)	4 (11.4)	5 (13.1)^2^
Prepubertal, n (%)	2 (3.8)	3 (5.2)	2 (4.4)	1 (2.8)	2 (5.3)
Pubertal, n (%)	5 (9.4)	5 (8.6)	4 (8.9)	3 (8.6)	3 (7.9)
Waist circumference, SDS	4.0 ± 1.4	3.9 ± 1.5	3.8 ± 1.1	4.1 ± 1.3	4.0 ± 1.3
Hip circumference, SDS	3.2 ± 1.0	3.3 ± 1.1	3.1 ± 1.3	3.2 ± 1.2	3.3 ± 1.2
Systolic BP, mmHg	115.9 ± 8.7	116.5 ± 8.8	118.2 ± 8.7	120.1 ± 7.9	119.8 ± 8.0
Diastolic BP, mmHg	68.2 ± 8.9	69.5.2 ± 8.9	69.4 ± 9.3	69.4 ± 8.6	69.9 ± 8.7
Fasting glucose, mg/dL	90.62 ± 4.32	91.23 ± 4.39	89.63 ± 3.17	90.90 ± 5.71	90.12 ± 5.79
Fasting insulin, μU/mL	27.60 ± 7.83	27.48 ± 7.92	26.71 ± 9.36	27.39 ± 8.61	27.46 ± 8.55
HbA1c, %, mmol/mol	5.63 ± 0.54	5.66 ± 0.57	5.70 ± 0.52	5.68 ± 0.58	5.69 ± 0.59
HOMA-IR, mean (range)	5.69 (3.60–9.92)	5.76 (3.60–10.67)	5.68 (2.00–10.90)	5.78 (4.21–8.89)	5.76 (3.21–8.89)
Prepubertal	5.61 (3.60–8.04)	5.72 (3.60–10.67)	4.72 (2.00–8.38)	5.32 (3.25–7.98)	5.32 (3.21–7.98)
Pubertal	7.41 (4.67–9.92)	7.39 (4.67–9.92)	6.31 (3.82–10.90)	7.19 (4.38–8.89)	7.19 (4.38–8.89)
IS_OGTT_, SDS	1.40 ± 0.24	1.42 ± 0.26	1.42 ± 0.22	1.56 ± 0.48	1.58 ± 0.49
Prepubertal	1.51 ± 0.15	1.52 ± 0.16	1.79 ± 0.55	1.56 ± 0.11	1.59 ± 0.15
Pubertal	1.27 ± 0.27	1.29 ± 0.31	1.35 ± 0.55	1.30 ± 0.22	1.31 ± 0.23
Insulinogenic index, mean (range)	3.66 (1.90–9.25)	3.61 (1.70–9.25)	2.54 (1.91–10.2)	3.12 (1.15–9.70)	3.07 (1.13–9.70)
Prepubertal	3.43 (2.29–9.25)	3.42 (2.29–9.25)	2.57 (1.29–10.26)	3.94 (2.51–9.70)	3.94 (2.51–9.70)
Pubertal	2.98 (1.90–6.36)	2.95 (1.70–6.36)	1.75 (0.63–4.30)	2.25 (1.15–3.24)	2.25 (1.13–3.29)
Disposition index, mean (range)	4.42 (2.25–11.97)	4.48 (2.22–12.02)	4.01 (0.83–11.72)	3.79 (1.88–9.28)	3.84 (1.88–14.78)
Prepubertal	5.52 (2.25–11.97)	5.55 (2.22–12.02)	4.59 (2.31–11.72)	5.68 (4.30–14.78)	5.69 (4.30–14.78)
Pubertal	4.42 (2.99–6.76)	4.44 (2.73–6.76)	2.48 (0.83–5.67)	2.72 (1.99–3.79)	2.79 (1.88–6.39)
Glucose metabolism abnormalities, n (%)	8 (15.1)	9 (15.5)	6 (13.3)	4 (11.4)	5 (11.4)
IGT	6 (11.3)	7 (12.1)^1^	5 (11.1)	4 (11.4)	4 (10.5)
T2DM	2 (3.8)	2 (3.4)	1 (2.2)	-	1 (2.6)^2^

BMI, body mass index; BP, blood pressure; HOMA-IR, homeostasis model of assessment for insulin-resistance; IS_OGTT_, Matsuda index; SDS, standard deviation score; IGT, impaired glucose tolerance; T2DM, type 2 diabetes mellitus; *per protocol analysis; °intention to treat analysis. ^1^patient with *acanthosis nigricans* and IGT dropped out in the per protocol analysis for non-compliance: ^2^patient with *acanthosis nigricans* and T2DM dropped out in the per protocol analysis for non-compliance.

Arm A comprised 53 subjects (n = 27 males, 26 females; median age, 11.7 years; age range, 8.0–13.6 years) who were treated with a LGI diet and Policaptil Gel Retard® (Aboca Spa Company, Sansepolcro, Arezzo, Italy). Arm B comprised 45 subjects (n = 23 males, 22 females; median age, 12.0 years; age range, 8.2–13.5 years) who were treated only with a LGI diet. Arm C comprised 35 subjects (n = 19 males, 16 females; median age, 11.8 years; age range, 8.1–13.1 years) who were treated only with an ERD of reduced carbohydrates and fats.

### Sample size calculation

The number of subjects required to compare the abilities of the three treatment arms to achieve a reduction in BMI of at least0.5 SDS and to detect a change of at least 10 μU/mL in basal insulin levels between the groups was calculated with a significance level of 5% and a power of 90%. The aim was to include at least a 1.3:1:1 ratio due to a higher withdrawal rate expected in Arm A.

### Study protocol

During the interventional study (T_0_ and T_1_), trained experts collected and assessed the clinical and demographic data, including height, weight, body mass index (BMI), pubertal stage, waist and hip circumference, and the time dedicated to outdoor physical activity. Furthermore, nutrient diaries were recorded for each subject based on his/her medical charts and standardised interviews. Blood samples (fasting and after glucose loading) were obtained in certified procedure rooms by certified paediatric nurses [17].

Obesity was defined based on the reference values in growth charts as shown in the study by Cacciari et al. [18] Children with a BMI greater than the 95th percentile for their age and gender were classified as obese. Those with BMIs equal to or exceeding the 85th percentile but below the 95th percentile were defined as overweight [19,20].

Policaptil Gel Retard® is the API (Active Pharmaceutical Ingredient) of the Medical Device in tablets, Libramed (Aboca Spa Company, Sansepolcro, Arezzo, Italy). This complex is composed of polysaccharidic macromolecules (cellulose, hemicellulose, pectin, mucilages) and is derived from the following raw materials rich in fibres: glucomannan (*Amorphophallus konjac*), cellulose, Opuntia pulp stem (*Opuntia ficus indica*), chicory root (*Cichorium intybus*), freeze-dried mallow root mucilage (*Althaea officinalis*), freeze-dried flaxseed mucilage (*Linum usitatissimum L*), and freeze-dried linden flower mucilage (*Tilia platyphyllos Scop*). This product slows the rate of carbohydrate absorption, likely decreasing glycaemic and insulinaemic peak intensity. The exact composition and the production process by which the API is obtained is covered by a European patent (n° 1679009).

All patients of Arm A took 3 tablets before their two main meals. Compliance was evaluated by means of written instructions provided at the onset of the study and at clinical controls through a written questionnaire completed by the parents. Compliance was verified by e-mails and telephone interviews performed by a study nurse (to confirm the use of Policaptil Gel Retard®) and by the bottle count performed at the end of the study period.

During the baseline and final medical evaluations, the parents completed a questionnaire that was reviewed by an interviewer and included items regarding the subject’s and his/her family’s lifestyle, such as detailed diet information, family and personal medical histories, and current medications [21]. During final evaluation a questionnaire was completed to assess any side effects that had occurred during the study.

The reported energy intake (REI) was calculated from 3-day diet records, typically two weekdays and one weekend day [22]. The families were provided with a sample food record with written and verbal instructions for their completion and 2-dimensional food models for estimating portions.

Daily energy requirements (DER) were calculated using the Schofield equation [23], which considers age, sex, height, weight, and physical activity. The REI/DER ratio was used to classify the intake as presumably under-reported (<0.8), adequately reported (0.8–1.2), or over-reported (>1.2) [24]. The physical activity levels were estimated according to the interview items based on the behavioural risk factor surveillance system [25].

The glycaemic index (GI) of a test food is defined as the glucose area under the curve (AUC) after consuming 50 g of carbohydrates of a test food relative to the value after consuming 50 g of carbohydrates of a standard food (either white bread or glucose) [26]. The GI of carbohydrate-containing foods was assigned on the basis of a glucose reference from published GI values [27]. A weighted GI for each food item was obtained by multiplying the GI by the proportion of total carbohydrates contributed by the food item. The daily GI was calculated by summing up the weighted GI values for each food item [27].

The subjects in the LGI diet groups, with or without the use of Policaptil Gel Retard®, were instructed to limit their intake of high GI foods (e.g., white bread and concentrated sugars) based on a food classification system by GI value, where ‘red,’ ‘yellow,’ and ‘green’ foods have high (≥70), medium (56–69), and low (≤55) GIs, respectively [28]. Green foods were unrestricted (e.g., fruit, non-starchy vegetables, 100% whole grains, and unbreaded meat, poultry and fish); yellow foods (e.g., pizza, macaroni and cheese, corn and dried fruits) were consumed less frequently; and red foods (e.g., sugary drinks, refined baked goods, candy, white bread and white potatoes) were restricted to ≤7 servings/week [28]. There were no specific restrictions on energy or fat intake for the LGI groups.

The ERD group was treated with the Traffic Light Diet to decrease energy, carbohydrate, and fat intake [29]. The Traffic Light Diet foods are categorised as ‘green,’ ‘yellow,’ and ‘red’ based on their fat and sugar content per serving. Green foods, such as fruits and vegetables, are high in nutrient density and low in energy density (fat = 0–1 g or sugar <10% calories/serving). Yellow foods are higher in energy density than green foods (fat = 2–5 g or sugar = 10–25% calories/serving). Red foods, such as the fat, oil, and sweet groups, are higher in energy density (fat >5 g or sugar >25% calories/serving). Modified foods from the fat, oil, and sweet groups are considered red foods, although their fat or energy level is low. However, combination foods (e.g., sandwiches, pizza, hamburger, and lasagne) are only considered red food if a serving contains at least one serving of a red food [29]. The ERD group was instructed to consume a distribution of macronutrients of 53% carbohydrates, 17% proteins, and 30% fat. The induced energy restriction was −30% with respect to the individually measured DER.

The variables for insulin resistance and β-cell function were evaluated using an oral glucose tolerance test (OGTT), carried out at baseline (T_0_) and at 12 months (T_1_). After an overnight fast of 12 h, the patients ingested 1.75 g of glucose per kilogram of body weight (maximal dose, 75 g), and their glucose and insulin levels were determined at baseline and 30 min, 60 min, 90 min, and 120 min later [17]. The glycaemic status was defined based on the 2010 American Diabetes Association criteria [30]: impaired fasting glucose, defined as a fasting plasma glucose (FPG) of 100–125 mg/dL (5.6–6.9 mmol/L) without hypoglycaemic treatment; impaired glucose tolerance (IGT), defined as 2-h values in the OGTT of 140–199 mg/dL (7.8–11.0 mmol/L); diabetes, defined as an FPG of ≥126 mg/dL (≥7.0 mmol/L) and 2-h plasma glucose values during an OGTT of ≥200 mg/dL (≥11.1 mmol/L).

The homeostasis model assessment of insulin resistance (HOMA-IR), Matsuda index of insulin sensitivity [31-33], and plasma glucose and insulin AUCs were calculated for all patients. A low HOMA-IR index indicated high insulin sensitivity, whereas a high HOMA-IR index indicated low insulin sensitivity (insulin resistance). An HOMA-IR index above 4.4 was considered to be consistent with insulin resistance [17,33]. The Matsuda index [32] provided a measure of insulin sensitivity and was calculated using the following equation:

$$ =\frac{10,000}{\sqrt{\left(\mathrm{F}\mathrm{P}\mathrm{G}\times \mathrm{F}\mathrm{P}\mathrm{I}\right)\times \left(\mathrm{mean}\ \mathrm{P}\mathrm{G}\times \mathrm{mean}\ \mathrm{P}\mathrm{I}\right)}} $$

where FPG is fasting plasma glucose, FPI is fasting plasma insulin, PG is plasma glucose, and PI is plasma insulin. The Matsuda index of insulin sensitivity is consistent with the direct measurements using an insulin clamp.

The glucose and insulin AUCs during the OGTT were calculated using the trapezoidal rule [34]. Delta-glucose (ΔG30–0) and delta-insulin (ΔI30–0) were evaluated as the changes in glucose and insulin concentrations from 0 min to 30 min. The insulinogenic index, calculated as (Ins30 - Ins0)/(Glu30 - Glu0), was used to estimate insulin secretion [35]. The β-cell compensatory capacity was evaluated using the disposition index (DI), defined as the product of the Matsuda and insulinogenic indexes [35].

For children aged 10 years and older, MetS was diagnosed by abdominal obesity and the presence of two or more clinical features [i.e., elevated triglycerides (TG), low high-density lipoprotein (HDL) cholesterol, high blood pressure, and elevated plasma glucose] [36].

## Methods

Height was measured to the nearest 0.1 cm in triplicate using a Harpenden stadiometer. Weight was determined to the nearest 0.1 kg using a standard physician’s beam scale with the subject shoeless and dressed in light underwear. BMI was calculated as body weight divided by height squared (kg/m^2^). The height, weight, and BMI were stratified using Italian growth charts [37].

Waist circumference was measured to the nearest 0.1 cm at the end of normal expiration using a non-elastic tape measure placed midway between the lowest rib margin and the iliac crest [38,39]. Hip circumference was measured to the nearest 0.1 cm using a tape measure positioned horizontally over the widest part of the gluteal region as the subject stood relaxed with his/her feet placed as close together as possible [38,39]. The waist/hip ratio was calculated using these measurements [40].

The height, weight, BMI, waist circumference, and hip circumference were normalised for chronological age by conversion to standard deviation scores (SDSs). Blood pressure was measured three times by trained personnel by auscultation using a mercury sphygmomanometer on the right arm after the patient has been sitting quietly for 5 minutes, with the back supported, feet on the floor, right arm supported and cubital fossa at heart level [17]. The mean systolic and diastolic values were recorded and stratified according to the paediatric percentiles of the National High Blood Pressure Education Program Working Group on High Blood Pressure in Children and Adolescents [17].

Serum glycaemia (Dimension RXL system, Dade Behring, Dallas, TX, USA) and serum insulin (IMMULITE 2000 analyser, Siemens Healthcare Diagnostics, Marburg, Germany) levels were measured using immunoenzymatic assays, and glycosylated haemoglobin (HbA1c) levels were determined using high performance liquid chromatography (DIAMAT, Bio-Rad, Richmond, CA, USA). The normal range for HbA1c was 4.2–6.0%, and the coefficient of variation (CV) was 4.8 at 5.5%.

The total cholesterol, HDL cholesterol, and triglyceride (TG) measurements were performed according to routine laboratory methods. Low-density lipoprotein (LDL) cholesterol was calculated using the Friedwald formula: LDL = total cholesterol − HDL cholesterol − TG/2.2 [17].

Hyperlipidaemia was defined as total cholesterol ≥5.0 mmol/L, LDL-cholesterol ≥3.5 mmol/L, or triglycerides ≥1.1 mmol/L for subjects younger than 10 years of age or ≥1.7 mmol/L for those older than 10 years of age. Serum HDL-cholesterol levels were considered to be low if they were <1.03 mmol/L in children older than 10 years if the values were <1.03 mmol/L in males or <1.29 mmol/L in females [17].

### Statistical analyses

The statistical analyses were performed using SPSSX software (SPSSX Inc., Chicago, IL, USA). The characteristics of the study population were described using frequency distributions for categorical variables and mean and standard deviation (SD) values, medians, and ranges for continuous variables, depending on whether the data were normally distributed. The differences between patient groups and controls were assessed using Student’s *t* test or the Mann–Whitney *U* test, depending on the distribution of the analysed variables. The chi-squared test and Fisher’s exact test were used to examine the associations between the dichotomous variables. The inter-group comparisons for parameters were conducted using analysis of variance (ANOVA) or repeated-measures analysis of covariance (ANCOVA), as appropriate. Bonferroni’s post hoc correction for multiple comparisons was also applied when a significant F was found. A p < 0.05 was considered to be statistically significant.

## Results

### One year interventional study: enrolment time or T_0_

The primary clinical and biochemical features of the study groups are shown in Table 1. At baseline we have not found differences in levels of physical activity, food and dietary habits of the different Arms (data not shown).

Mean BMI-SDS was 2.29 ± 0.58 (2.18 ± 0.53 in prepubertal subjects and 2.31 ± 0.63 in pubertal subjects), without differences between males (2.31 ± 0.70) and females (2.19 ± 0.46). Seventeen patients (12.8%, n = 9 males and 8 females; median age, 12.5 years; age range, 8.8–13.2 years) had *acanthosis nigricans*. However, eighteen (13.5%) patients showed glucose metabolism abnormalities: 15 (11.3%) patients showed IGT, and 3 (2.2%) exhibited T2DM. BMIs were not significantly different between the patients with glucose metabolism abnormalities (2.31 ± 0.61) and without glucose metabolism abnormalities (2.19 ± 0.57).

At T_0_, the median HOMA-IR values were 5.69 (range, 3.60–9.92), 5.68 (range, 2.00–10.90), and 5.78 (range, 4.21–8.89) for Arms A-C, respectively. Insulin sensitivity was reduced (1.40 ± 0.24, 1.42 ± 0.22; and 1.56 ± 0.48 in Arms A-C, respectively). The insulinogenic indices for Arms A-C were 3.66 (1.90–9.25), 2.54 (1.91–10.2), and 3.12 (1.15–9.70), respectively, and the disposition indices were 4.42 (2.25–11.97), 4.01 (0.83–11.72), and 3.79 (1.88–9.28). Regarding these indices we have not found any significant differences between males and females, whereas we discovered significant statistical differences between prepubertal and pubertal subjects.

There were no significant differences among Arms A-C in the total cholesterol level (4.47 ± 0.74 mmol/L; Arms A-C: 4.39 ± 0.51, 4.36 ± 1.09, and 4.51 ± 0.79 mmol/L, respectively), mean LDL cholesterol (3.05 ± 0.60, 2.96 ± 0.73, and 3.12 ± 0.81 mmol/L, respectively), or triglycerides (1.54 ± 0.40, 1.48 ± 0.29, and 1.62 ± 0.51 mmol/L, respectively).

### One year interventional study: end time or T_1_

The comparison between clinical and biochemical features of the study groups are shown in Tables 2 and 3.

**Table 2:** **Comparison of clinical and biochemical variables at baseline and at the end of the study in the different arms (per protocol analysis)**

	**Arm A**		**Arm B**		**Arm C**	
	**T**_**0**_	**T**_**1**_	**T**_**0**_	**T**_**1**_	**T**_**0**_	**T**_**1**_
BMI (SDS)	2.32 ± 0.53	1.80 ± 0.36***	2.23 ± 0.57	1.99 ± 0.56*°	2.27 ± 0.74	2.18 ± 0.70°°°
*Acanthosis nigricans*, n (%)	7 (13.2)	3 (5.6)**	6 (13.3)	6 (13.3)	4 (11.4)	4 (11.4)
IGT	6 (11.3)	4 (7.5)	5 (11.1)	4 (8.8)	4 (11.4)	3 (8.6)
T2DM	2 (3.8)	1 (1.9)	1 (2.2)	1 (2.2)	-	-
Fasting glucose, mg/dL	90.62 ± 4.32	84.02 ± 4.23***	89.63 ± 3.17	86.72 ± 6.00^§§§^°°°	90.90 ± 5.71	90.12 ± 2.89°°°
Fasting insulin, μU/mL	27.60 ± 7.83	18.04 ± 3.44***	26.71 ± 9.36	22.09 ± 3.67**°°°^§§§^	27.39 ± 8.61	25.91 ± 4.41°°°
HbA1c, %, mmol/mol	5.63 ± 0.54	5.37 ± 0.35**	5.70 ± 0.52	5.40 ± 0.51*	5.68 ± 0.58	5.54 ± 0.50
HOMA-IR, mean (range)	5.69 (3.60–9.92)	3.38 (2.64–5.38)***	5.68 (2.00–10.90)	4.90 (3.15–6.26)*°°°^§§§^	5.78 (4.21–8.89)	5.76 (4.00–6.19)°°°
IS_OGTT_, SDS	1.40 ± 0.24	3.16 ± 0.83***	1.42 ± 0.22	1.74 ± 0.40***°°°	1.56 ± 0.48	1.43 ± 0.21°°°
Insulinogenic index, mean (range)	3.66 (1.90–9.25)	2.79 (1.84–8.27)*	2.54 (1.91–10.2)^##^	2.40 (0.82–5.87)	3.12 (1.15–9.70)	2.95 (1.22–6.78)
Disposition index, mean (range)	4.42 (2.25–11.97)	10.28 (5.36–21.65)***	4.01 (0.83–11.72)	4.93 (1.53–7.27)*°°°	3.79 (1.88–9.28)	3.84 (1.69–5.82)°°°
Total cholesterol (mmol/L)	4.39 ± 0.51	3.31 ± 0.59***	4.36 ± 1.09	3.58 ± 0.93**	4.51 ± 0.79	3.74 ± 0.86**°
LDL cholesterol (mmol/L)	3.05 ± 0.60	2.87 ± 0.67	2.96 ± 0.73	2.92 ± 0.78	3.12 ± 0.81	2.99 ± 0.87
Triglycerides (mmol/L)	1.54 ± 0.40	1.63 ± 0.45	1.48 ± 0.29	1.61 ± 0.43	1.62 ± 0.51	1.54 ± 0.58

BMI, body mass index; BP, blood pressure; HOMA-IR, homeostasis model of assessment for insulin-resistance; IS_OGTT_, Matsuda index; SDS, standard deviation score; IGT, impaired glucose tolerance; T2DM, type 2 diabetes mellitus. Cross-sectional study: Arm A *vs.* Arm B or Arm A *vs.* Arm C: ^##^p < 0.005. Longitudinal study: Arm A *vs.* Arm A. Arm B *vs.* Arm B, Arm C *vs.* Arm C: *p<0.05, **p<0.005, ***p < 0.0005; Arm A *vs.* Arm B or Arm A *vs.* Arm C: °p < 0.05, °°°p < 0.0005; Arm B vs. Arm C: ^§§§^p < 0.0005.

**Table 3:** **Comparison of clinical and biochemical variables at baseline and at the end of the study in the different arms (intention to treat analysis)**

	**Arm A**		**Arm B**		**Arm C**	
	**T**_**0**_	**T**_**1**_	**T**_**0**_	**T**_**1**_	**T**_**0**_	**T**_**1**_
BMI (SDS)	2.33 ± 0.57	1.89 ± 0.45***	2.23 ± 0.57	1.99 ± 0.56^*^°	2.29 ± 0.77	2.21 ± 0.73°°°
*Acanthosis nigricans*, n (%)	8 (13.8)^1^	3 (5.1)***	6 (13.3)	6 (13.3)	5 (13.1)^2^	4 (10.5)
IGT	7 (12.1)^1^	4 (6.9)*	5 (11.1)	4 (8.8)	4 (10.5)	3 (8.6)
T2DM	2 (3.4)	1 (1.7)	1 (2.2)	1 (2.2)	1 (2.6)^2^	1 (2.6)
Fasting glucose, mg/dL	91.23 ± 4.39	86.34 ± 4.69***	89.63 ± 3.17	86.72 ± 6.00^§§§^	90.12 ± 5.79	91.36 ± 2.98°°°
Fasting insulin, μU/mL	27.48 ± 7.92	20.23 ± 5.12***	26.71 ± 9.36	22.09 ± 3.67**^§§§^	27.46 ± 8.55	25.32 ± 4.19°°°
HbA1c, %, mmol/mol	5.66 ± 0.57	5.43 ± 0.39**	5.70 ± 0.52	5.40 ± 0.51*	5.69 ± 0.59	5.54 ± 0.50
HOMA-IR, mean (range)	5.76 (3.60–10.67)	3.73 (2.64–7.13)**	5.68 (2.00–10.90)	4.90 (3.15–6.26)*°°^§§^	5.76 (3.21–8.89)	5.63 (3.11–6.37)°°
IS_OGTT_, SDS	1.42 ± 0.26	3.01 ± 0.85***	1.42 ± 0.22	1.74 ± 0.40***°°	1.58 ± 0.49	1.43 ± 0.21*°°°
Insulinogenic index, mean (range)	3.61 (1.70–9.25)	2.86 (1.84–6.78)*	2.54 (1.91–10.2)^##^	2.40 (0.82–5.87)	3.07 (1.13–9.70)	2.82 (1.13–7.05)
Disposition index, mean (range)	4.48 (2.22–12.02)	9.13 (5.01–21.65)***	4.01 (0.83–11.72)	4.93 (1.53–7.27)*°°°	3.80 (1.88–14.78)	4.14 (1.42–5.98)°°°
Total cholesterol (mmol/L)	4.38 ± 0.52	3.63 ± 0.68***	4.36 ± 1.09	3.58 ± 0.93**	4.49 ± 0.80	3.67 ± 0.62**
LDL cholesterol (mmol/L)	3.17 ± 0.64	2.97 ± 0.71	2.96 ± 0.73	2.92 ± 0.78	3.17 ± 0.83	3.03 ± 0.94
Triglycerides (mmol/L)	1.58 ± 0.43	1.88 ± 0.49	1.48 ± 0.29	1.61 ± 0.43°°	1.68 ± 0.53	1.51 ± 0.67°°

BMI, body mass index; BP, blood pressure; HOMA-IR, homeostasis model of assessment for insulin-resistance; IS_OGTT_, Matsuda index; SDS, standard deviation score; IGT, impaired glucose tolerance; T2DM, type 2 diabetes mellitus. ^1^patient with *acanthosis nigricans* and IGT dropped out in the per protocol analysis for non-compliance: ^2^patient with *acanthosis nigricans* and T2DM dropped out in the per protocol analysis for non-compliance. Cross-sectional study: Arm A *vs.* Arm B or Arm A *vs.*Arm C: ^##^p < 0.005. Longitudinal study: Arm A *vs.* Arm A. Arm B *vs.* Arm B, Arm C *vs.* Arm C: *p < 0.05, **p < 0.005, ***p < 0.0005; Arm A *vs.* Arm B or Arm A *vs.* Arm C: °p < 0.05, °°p < 0.005, °°°p < 0.0005; Arm B vs. Arm C: ^§§^p < 0.005, ^§§§^ p < 0.0005.

The use of Policaptil Gel Retard® has proved extremely safe. In fact, there was evidence of only sporadic and mild side effects represented by abdominal swelling (1 patients, 0.75%), feeling of abdominal distension (2 patients, 1.5%), flatulence (2 patients, 1.5%), and diarrhoea (1 patient, 0.75%). These effects were transient, and no patient had to discontinue the study.

**Figure 2:**
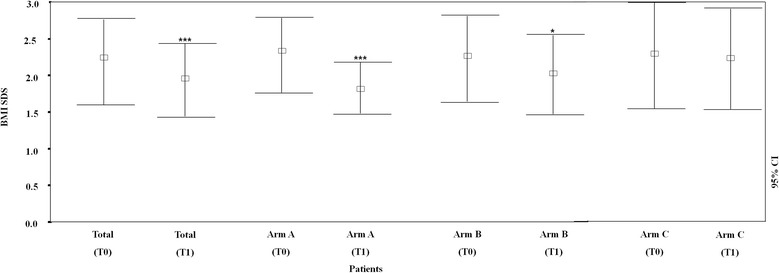
**Basal and longitudinal comparison of body mass index (BMI) SDS of all of the patients in Arm A, Arm B, and Arm C.** The bars represent mean values and 95% confidence intervals. *p < 0.05; ***p < 0.001.

There were statistically significant differences in weight reduction among the arms at T_1_ (Figure 2). The mean BMI-SDS was 1.94 ± 0.50, significantly less than T_0_ (2.29 ± 0.58; P < 0.0001). For Arms A-C, the BMI-SDSs were 1.80 ± 0.36 (T_0_: 2.32 ± 0.53; p < 0.0001), 1.99 ± 0.56 (T_0_: 2.23 ± 0.57; p < 0.05), and 2.18 ± 0.70 (T_0_: 2.27 ± 0.74; insignificant), respectively. Arm A subjects have significantly reduced BMI-SDSs compared to Arm B (p < 0.05) and Arm C subjects (p < 0.0001).

We did not observe a significant reduction of patients with *acanthosis nigricans* (9.8% *vs.* 12.8%; p = NS): however, the number declined significantly from 7 to 3 (5.6% *vs.* 13.2%; p < 0.005) in Arm A and not change significantly in Arms B (13.3%) and C (11.4%). However, we observed a significant reduction in HbA1c with respect to the T_0_ (5.36 ± 0.44% *vs.* 5.64 ± 0.53%, p < 0.0001). This reduction was significant in Arm A (5.37 ± 0.35% *vs.* 5.63 ± 0.54%; p < 0.005) and B (5.40 ± 0.51% *vs.* 5.70 ± 0.52%; p < 0.05), but not in Arm C (5.54 ± 0.50% *vs.* 5.68 ± 0.58%).

Overall, there was a reduction in patients showing glucose metabolism abnormalities (9.8% *vs.* 13.5%: 8.3% have IGT, whereas 1.5% have T2DM). IGT was diagnosed in 7.5% (*vs.* 11.3%), 8.8% (*vs.* 11.1%), and 8.6% (*vs.* 11.4%) of all patients in Arms A-C, respectively, whereas T2DM was diagnosed in 1 patient in Arms A (1.9% *vs.* 3.8%) and B (2.2% *vs.* 2.2%). BMI was not significantly different in patients with glucose metabolism abnormalities (1.93 ± 0.49) and without glucose metabolism abnormalities (1.95 ± 0.57), even if we showed a significant difference in respect to T_0_ in patients with glucose metabolism abnormalities (2.31 ± 0.61; p < 0.05), but not in those without glucose metabolism abnormalities (2.19 ± 0.57).

**Figure 3:**
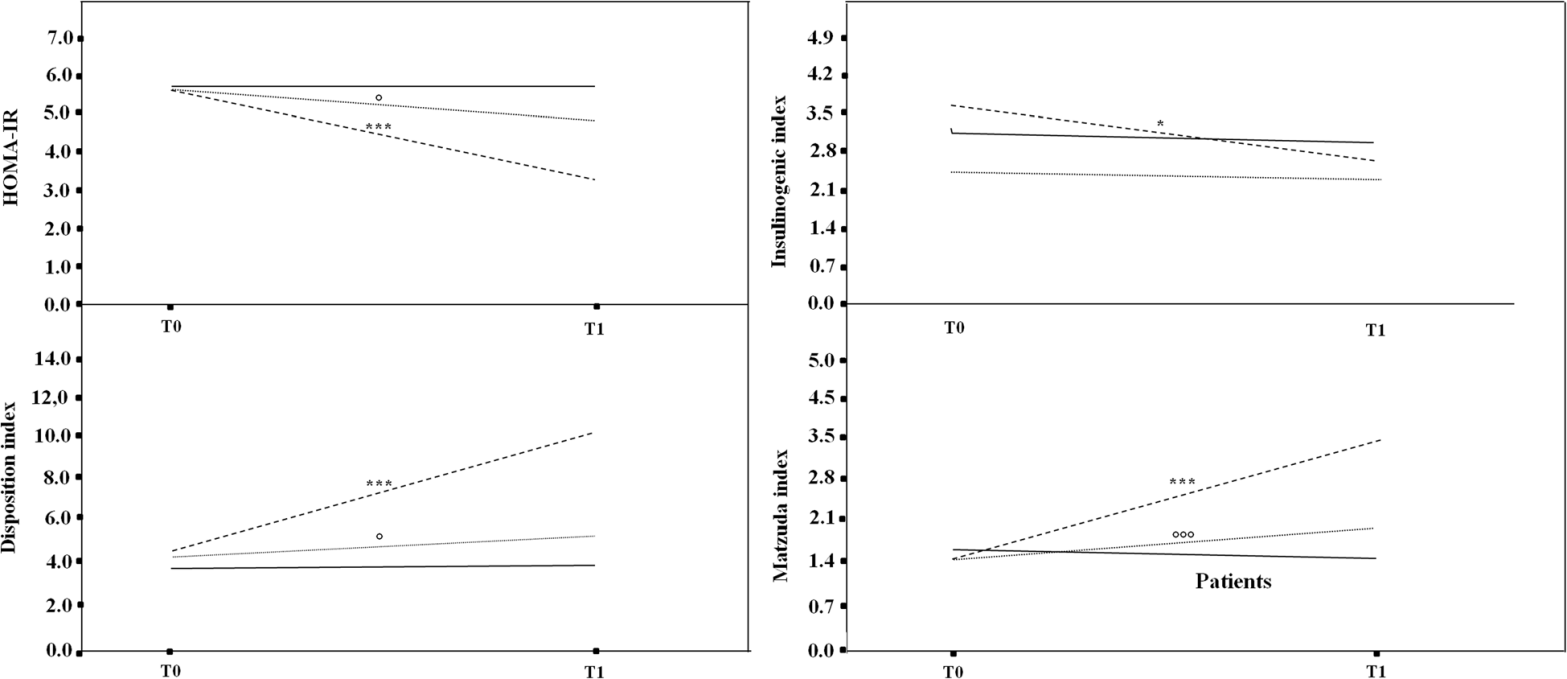
**Basal and longitudinal comparison among the three arms (Arm A, broken line; Arm B, dotted line; Arm C, solid line) regarding HOMA-IR, insulinogenic index, disposition index, and Matsuda index.** The bars represent mean values and 95% confidence intervals. *Arm A: *p < 0.05; **p < 0.005; ***p < 0.001. °Arm B: °p < 0.05; °°p < 0.005; °°°p < 0.0005. ^Arm C 3: ^p < 0.05; ^^p < 0.005; ^^^p < 0.0005.

The median HOMA-IR was significantly less than T_0_ in Arms A (3.38; range, 2.64–5.38; p < 0.0001) and B (4.90; range, 3.15–6.26; p < 0.05) but not C (5.76; range, 4.00–6.19). Insulin and glucose levels were significantly reduced in Arm A (18.04 ± 3.44 μU/mL *vs.* 27.60 ± 7.83 μU/mL, p < 0.0001; 84.02 ± 4.23 mg/dL *vs.* 90.63 ± 4.32 mg/dL, p < 0.0001, respectively), whereas in Arm B, we observed a reduction in insulin (22.09 ± 3.67 μU/mL *vs.* 26.71 ± 9.36 μU/mL, p < 0.005) but not in glucose levels (86.72 ± 6.00 mg/dL *vs.* 89.63 ± 3.17 mg/dL). In Arm C, insulin and glucose levels were not different (25.91 ± 4.41 μU/mL and 90.12 ± 2.89 mg/dL, respectively) in respect to T_0_ (Figure 3).

The insulin sensitivity was significantly ameliorated in Arm A (3.16 ± 0.83 *vs.* 1.40 ± 0.24, p < 0.0001) and B (1.74 ± 0.40 *vs.* 1.42 ± 0.22, p < 0.0001), with a significant difference between the two arms (p < 0.0001). The insulin sensitivity in Arm C did not change significantly (1.43 ± 0.21 *vs.* 1.56 ± 0.48) (Figure 3).

Arm A showed a significant reduction in insulinogenic index (2.79 [1.84–8.27] *vs.* 3.66 [1.90–9.25], p < 0.05). Arms B and C had reductions from 2.54 (1.91–10.2) to 2.40 (0.82–5.87) and from 3.12 (1.15–9.70) to 2.95 (1.22–6.78), respectively, which were not significant (Figure 3).

The disposition index was significantly higher in Arm A [10.28 (5.36–21.65) *vs.* 4.42 (2.25–11.97) at T_0_; p < 0.0001] and Arm B [4.93 (1.53–7.27) *vs.* 4.01 (0.83–11.72) at T_0_; p < 0.05]; however, there was no significant change in Arm C [3.84 (1.69–5.82) *vs.* 3.79 (1.88–9.28) at T_0_] (Figure 3).

The mean total cholesterol was 3.73 ± 0.89 mmol/L (Arms A-C: 3.31 ± 0.59 mmol/L, 3.58 ± 0.93 mmol/L, and 3.74 ± 0.86 mmol/L, respectively). The LDL cholesterol was 2.87 ± 0.67 mmol/L in Arm A, 2.92 ± 0.78 mmol/L in Arm B, and 2.99 ± 0.87 mmol/L in Arm C, respectively, not significantly reduced in respect to T_0_. Finally, the triglyceride levels were 1.63 ± 0.45 mmol/L in Arm A, 1.61 ± 0.43 mmol/L in Arm B, and 1.54 ± 0.58 mmol/L in Arm C, respectively, not significantly reduced with respect to T_0_.

## Discussion

Our study confirms that weight reduction is more efficient in individuals subjected to a LGI carbohydrate diet compared to an ERD. Interestingly, Policaptil Gel Retard® significantly potentiated the effectiveness of the LGI diet in reducing BMI. However, its use in conjunction with a LGI diet, significantly reduced the frequency of *acanthosis nigricans* and the HbA1c levels in these patients. Furthermore, our data suggest that Policaptil Gel Retard® may reduce the number of glucose metabolism abnormalities, such as IGT and T2DM in obese patients. Although the differences are not significant, our results potentially highlight a new interesting effect of this polysaccharidic macromolecules complex.

In fact, this effect of Policaptil Gel Retard® may be related to a reduction in the post-meal glycaemic and insulinaemic peaks. The attenuated pancreatic insulin response was likely due to slow glucose absorption, given that glucose absorption directly regulates pancreatic insulin release [41].

Both the quantity and the quality of a carbohydrate affect the postprandial glycaemia and the interaction between the two appears to be synergistic. Carbohydrates affect energy intake and body weight by their GI, and different carbohydrate foods can increase glucose and insulin levels to varying degrees, even when the amount consumed is similar [41]. A high-carbohydrate diet based on high GI foods is digested and absorbed rapidly, resulting in high glycaemic load and increased demand for insulin secretion [42]. In insulin-resistant people who consume high GI foods, the postprandial hyperglycaemia and insulinaemia are magnified, likely contributing to β-cell exhaustion and the development of T2DM [42].

The changes in glucose and insulin levels may have subsequent effects on food intake or may promote weight gain and obesity [40]. Typically, a low GI diet produces greater satiety, directly provoking greater increases in cholecystokinin and post-meal fullness/satiation [42-45]. In contrast, the rapid absorption of glucose from a high GI meal may increase voluntary food intake by rapid increases in glycaemia and insulinaemia in the postprandial period, leading to a reactive hypoglycaemia that stimulates appetite and increases the daily caloric intake [42,46].

Our data seem to suggest that Policaptil Gel Retard® may help reduce insulinemic peaks, enhancing β-cell function more effectively than the LGI diet alone does, and particularly the ERD, which seems to be unable to restore the insulin secretory reserve in patients with IGT or T2DM. However, taking into account that IGT is unstable, particularly in adolescents, long-term and more large-scale trials are required before more definitive statements can be made. Anyway, it is interesting to note that our data at baseline on the total of our patients and on 3 Arms after randomization are very similar to those reported by Brufani et al. with a cross-sectional study of 510 overweight and obese subjects aged 3–18 yr [47].

In the last decade, T2DM has emerged as an increasingly common paediatric disease in Europe [10] and studies have demonstrated that IGT in obese youths is associated with severe insulin resistance, β-cell dysfunction, and altered abdominal and muscle fat partitioning [48,49]. Obesity, which has become a problem in paediatrics, is among the major risk factors for the development of T2DM in children with a strong family history for the disease [48].

Although the transition from normoglycaemia to IGT and subsequently to diabetes in adults is usually a gradual phenomenon that occurs over 5–10 years, the early appearance/onset of T2DM in young people raises the likelihood of an accelerated process that shortens the transition time between these steps [48]. Thus, T2DM in young people has emerged as a paediatric entity of great concern in its own right throughout the developed world [48].

Weiss et al. [48] showed that IGT in obese children was a pre-diabetic transitional state to T2DM and that the primary predictor for deterioration to T2DM was the BMI z-score [48].

The role of the LGI diet, particularly in association with Policaptil Gel Retard®, may be important for reducing or slowing the progression from IGT to T2DM in obese children. Many studies have shown that very low carbohydrate diets, although they are ad libitum, seem to be more efficacious than energy-restricted, low fat diets over the short term [50-52], even in adults with T2DM [52]. This finding was confirmed in our study with regard to weight reduction and the amelioration of glycaemic and insulinemic metabolism as shown by the disposition index. Whereas at baseline the alterations in insulin sensitivity were not correctly compensated for by increases in the insulin response to glucose loading, the end-of-study disposition index was a significant predictor of amelioration for those who received the LGI diet that included Policaptil Gel Retard®. This observation may be important because the diet may slow the rate of deterioration of β-cell function in obese children, a rate that has been demonstrated to be faster in children than in adults [48].

Nevertheless, the relatively small sample size may be a limit of our study. Furthermore, although the data allow to hypothesize an effect of Policaptil Gel Retard® for ameliorating the glucoinsulinaemic metabolism, we cannot even exclude a concomitant effect of weight loss in these patients. A placebo group with a LGI diet alone or a group treated with only Policaptil Gel Retard® and normal diet would be useful to better distinguish the effects of this product.

## Conclusions

Policaptil Gel Retard® may be useful for reducing weight gain in obese children, particularly in conjunction with a LGI diet. It may also be useful for ameliorating the glucoinsulinaemic metabolism, reducing the use of future pharmacological interventions. Nevertheless, our results should be confirmed with additional studies that include larger sample sizes and longer longitudinal follow-up times.
